# Tomato Disease Combat and Yield Optimization Using Selenium Nanoparticles: A Sustainable Nano‐Agriculture Approach

**DOI:** 10.1002/fsn3.70386

**Published:** 2025-06-27

**Authors:** Zainab Maqbool, Arusa Aftab, Humaira Rizwana, Zubaida Yousaf, Shazia Rehman

**Affiliations:** ^1^ Department of Botany Lahore College for Women University Lahore Pakistan; ^2^ Department of Botany and Microbiology King Saud University Riyadh Saudi Arabia; ^3^ Sanya Nanfan Research Institution/College of Tropical Crops Hainan University Sanya China

**Keywords:** microbial pathogens, nanotechnology, pesticide, SeNPs, tomato, *Tuta absoluta*

## Abstract

Tomatoes are a crucial component of the global food system but are susceptible to pests and diseases, such as leaf miner (*Tuta absoluta*), fungal, and bacterial infections, especially in Pakistan. Synthetic pesticides pose risks to the environment and human health. This study aimed to explore the effectiveness of selenium nanoparticles (SeNPs) in combating these devastating pathogens. SeNPs were synthesized using citrus fruit peel essential oil extracted through hydro‐distillation and characterized through X‐ray diffraction (XRD) and UV–visible spectrometry. The optimal dosage of SeNPs was determined through bioactivities like antioxidant, antimicrobial, anti‐inflammatory, and pesticidal activity before seed priming. UV–visible spectroscopy revealed a surface plasmon resonance at 265 nm. XRD analysis confirmed a crystalline size of 6.4 nm for SeNPs. FTIR analysis confirmed the presence of potential functional groups in the essential oils mediated SeNPs, including alkanes, alkenes, alkyl halides, amines, aromatic compounds, isothiocyanate, alcohols, and carboxylic acids. The synthesized SeNPs demonstrated antimicrobial efficacy against common tomato‐infecting pathogens like *Fusarium oxysporum, Aspergillus niger, Pseudomonas syringae,* and *Xanthomonas campestris.* Against both bacterial and fungal strains, 10 ppm SeNPs showed a significant diameter of the zone of inhibition, i.e., 39.3 and 28.1 mm against *P. syringae* and *X. campestris* , and 26.3 and 39 mm against *A. niger* and *F. oxysporum*. SeNPs also exhibited significant pesticidal potential against *Tuta absoluta*. SeNPs enhanced seed germination percentage up to 94.8%, chlorophyll stability index (84.6%), membrane stabilization index (94%), and reduced root ion leakage (0.183%) at *p* < 0.001. The present study optimizes the dosage of SeNPs, which shows potential for antimicrobial and pesticidal efficacy, as well as improving seed germination rate, seedling vigor index, chlorophyll stability index, membrane stabilization index, and reducing root ion leakage. These findings offer new insights for sustainable crop management.

## Graphical abstract

This study aimed to explore the effectiveness of Selenium nanoparticles (SeNPs) in combating the devastating pathogens of tomato crop. The study also optimized the dosage of SeNPs, for potential antimicrobial and pesticidal efficacy.

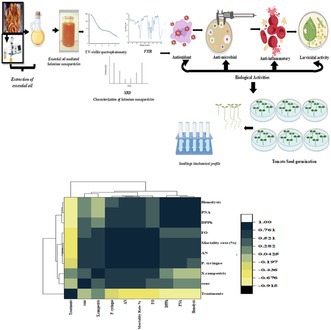

**Abbreviations**
*β*
: full width at half‐maximum
*λ*
: wavelength
BSA: Bovine serum albumin
CRBD: completely randomized Block design
CSI: chlorophyll stability index
DPPH: 2,2‐diphenyl‐1‐picrylhydrazyl
EC: electrical conductivity
EO: essential oil
FFCB‐IAGS: First Fungal Culture Bank Institute of Agricultural Sciences
FTIR: Fourier Transform Infrared Spectrometry
FW: fresh weight
FWHM: full width at half‐maximum
GC–MS: gas chromatography–mass spectrometry
GI: germination index
GP: germination percentage
HATR: horizontal attenuated total reflectance accessory
HRBC: human red blood cells
K: Scherrer constant
KBr: potassium bromide
MGT: mean germination time
MSI: membrane stability index
Na_2_SeO_3_
: sodium selenite
NaCl: sodium chloride
NIST: National Institute of Standards and Technology
ns: nonsignificant
PBS: phosphate‐buffered saline
SeNPs: selenium nanoparticles
SVI: seedling vigor index
T50: days to 50% germination
UV: Ultraviolet
XRD: X‐ray diffractometer

## 1 Introduction

Tomato ( *Lycopersicon esculentum* Mill) is globally recognized as one of the most important vegetable crops. Its fruits are rich in essential vitamins, nutrients, minerals, and pigments, such as lycopene (Lu et al. 2019; Helyes et al. 2009). Tomato fruits are consumed as fresh fruit, either cooked, raw, or in various other processed forms. They are a major source of carotenoids and many other health‐promoting bioactive compounds, playing an important role in human health and nutrition (Meng et al. 2022). Tomatoes grow well at temperatures between 26°C and 29°C and require a relative humidity of 65%–85% (Harel et al. 2014). China leads in tomato production with an annual output of 64,865,807 t, followed by India (20,573,000 t), Turkey (13,204,015 t), USA (12,227,402 t), Egypt (6,731,220 t), Italy (6,247,910 t), Iran (5,787,094 t), Spain (4,312,900 t), Mexico (4,317,342 t), and Brazil (3,753,595 t) (FAOSTAT 2022; Harel et al. 2014; Helyes et al. 2009). According to the FAO, global tomato production in 2022 was 186.82 million tonnes from a cultivation area of 5 million hectares, with a productivity of 36.97 t/ha (Govindasamy et al. 2025; Prasanna et al. 2023). According to the World Processing Tomato Council (2024), global tomato production decreased from 52.1 million tons to 50.9 million short tons, attributed to rising temperatures, insect attacks, and microbial pathogens (WPTC 2024; Attia et al. 2022; Tijjani et al. 2014; Negi et al. 2018). Agriculture is the backbone of Pakistan's economy, contributing only 0.3% of the world's tomato supply. Pakistan ranked 14th among the top tomato importers (Cherif and François 2019; Qasim et al. 2018; Negi et al. 2018). Despite its worldwide significance, tomato production faces significant challenges in various regions of the world, like Pakistan. *Tuta absoluta* (leaf miner), seed‐born fungal disease (*Fusarium oxysporum* f.sp. *lycopersici*), and bacterial infections ( *Xanthomonas campestris* ) are among the main pathogens responsible for the decline in tomato production in major growing regions of Pakistan (Chohan et al. 2017; Gabol et al. 2023). *Tuta absoluta* (Meyrick) is an invasive pest that negatively affects solanaceous crops (potato, tomato, and brinjal) (Akbar et al. 2018). *T. absoluta* larvae feed on leaves, young stems, and fruits, and adults lay eggs on leaves or young stems. This feeding habit threatens tomato production, potentially causing yield losses of 80%–100% (Ahmad and Ahmad 2022; Firdous 2021). The ideal temperature for *T. absoluta* growth and development is between 25°C and 30°C (Silva et al. 2021; Pandey et al. 2023). *Xanthomonas campestris* is a highly destructive microbe that infects tomato plants, leading to bacterial spot disease. It affects various parts of the plant, including stems, fruits, leaves, and flowers, resulting in significant crop losses (Oliveira et al. 2009). Bacterial speck disease in tomatoes is caused by *Pseudomonas syringae* (Andow 2024). Numerous fungal diseases have been documented in tomato fruit, such as *Rhizopus* rot, which is caused by *Rhizopus stolonifer, Aspergillus* rot, which is caused by *Aspergillus niger* (Negi et al. 2024; Zang et al. 2022), and *Fusarium* rot, which is caused by *F. oxysporum*. Infection caused by *F. oxysporum* harms crops, especially vegetables. *F. oxysporum*, the causative agent of fusarium wilt disease, seriously damages tomatoes at every stage of growth (Ebele 2011; Wakil et al. 2024).

Diamides have been used to treat tomato pinworms. Various approaches are used to manage microbial diseases in tomatoes, including planting resistant cultivars, maintaining good sanitation practices, using pathogen‐free seeds, and applying chemical controls like streptomycin and copper sprays (Teixeira and Andaloro 2013). Regular application of synthetic antimicrobial solutions and insecticides can lead to resistance, emphasizing the need for new insecticides, fungicides, and bactericides tailored to combat tomato pests effectively. Failure to introduce new active components in tomato disease control may result in decreased effectiveness and resistance against pests and pathogens (Abd El‐Aziz et al. 2019). Consequently, there has been a trend of increasing the concentration of traditional fertilizers to improve crop quality and yield. However, the excessive use of fertilizers over an extended period has led to numerous international environmental issues (Chi et al. 2022; Salama et al. 2022). A major portion of commercial fertilizers or pesticides applied to plants accumulates in water bodies and soil through leaching or mineralization processes (Nongbet et al. 2022). In response to these challenges, scientists worldwide are exploring alternative fertilizers to increase crop production while minimizing environmental hazards (Chhipa 2017). Despite remarkable advancements in agriculture production globally, food security and environmental sustainability remain significant challenges (Bisht and Singh 2021). Conventional approaches are proving insufficient to meet the growing needs, prompting the exploration of nanotechnology as a potential solution to expedite crop yield, production, and nutritional value while maintaining environmental sustainability (Hofmann et al. 2020). Nanoparticles, ranging from 1 to 100 nm in size, offer unique physical, chemical, and biological properties due to their high surface‐to‐volume ratio. Nanoparticle‐assisted food processing and packaging have transformed food systems. Novel nanoparticles have demonstrated potential in degrading foodborne pathogens (Ravichandran 2010). The application of optimal nanoscale concentrations of essential nutrients has resulted in significant improvements in seed germination, plant nutrition quality, and yield. Zinc oxide‐coated active packaging enhances the shelf life of fresh‐cut Fuji apples as a fresh‐cut product (Li et al. 2011). Nanoencapsulation of food products helps retain and enhance flavor (Nakagawa 2014). Green synthesis of nanoparticles provides a non‐toxic, eco‐friendly, and cost‐effective approach that is easy to implement and reproducible without the need for high‐tech equipment (Nasrollahzadeh et al. 2019). Essential oils (EO), aromatic compounds found in fruit peels and other plant parts, have attracted attention for their bioactive properties (Sganzerla et al. 2023). Selenium (Se) is a vital micronutrient essential for human health, playing a crucial role in various biological functions through selenocysteine integration (Ibrahim et al. 2019; Thiry et al. 2012). Fruits and vegetables are primary dietary sources of selenium, with the World Health Organization recommending a daily intake of 50–55 μg for humans (WHO 2009). Selenium uptake by plants helps protect them from herbivores and pathogens (Pilon‐Smits 2014; Zhang and Chu 2022). While excess selenium can be toxic to plants, it plays a crucial role at lower concentrations, with organic and inorganic selenium compounds commonly used as fertilizers (Pouri et al. 2018). Selenium nanoparticles are gaining attention and are widely used in electronics, food, medicines, and optics. Nanosized selenium exhibits excellent biological activities with low toxicity. Selenium nanoparticles have shown antibacterial efficacy against *Bacillus cereus, Listeria innocua, Salmonella typhimurium*, and *Escherichia coli* (Ndwandwe et al. 2021; Hussain et al. 2023).

Citrus EO, rich in volatile and non‐volatile compounds, has been shown to inhibit microbial growth due to its lipolytic moieties (Preedy 2015; Mahato et al. 2019). Citrus EO, primarily extracted from discarded fruit peels, offers a sustainable solution to reduce environmental pollution (Mahato et al. 2019). In the present study, Citrus fruit peel EO was extracted using the hydro‐distillation method. Selenium nanoparticles were synthesized using the EO, and the efficacy of EO‐mediated SeNPs in the germination of tomato seeds was assessed after biological activities.

## 2 Materials and Methods

### 2.1 Materials and Reagents

In this study, acetone, anhydrous sodium sulfate, alpha‐tocopherol, bovine serum albumin (BSA), dichloromethane, ethanol, methanol, phosphate‐buffered saline (PBS), sodium selenite, Diclofenac Sodium, and Flubendiamide were used. All the chemicals were purchased from Falcon Scientific, Lahore, originating from Merck, Germany, and Sigma Aldrich, USA.

### 2.2 Collection of Citrus Fruit Peels

Fresh orange ( *Citrus sinensis* ) fruits were collected from the citrus research institute Sargodha, Punjab, Pakistan. The peels were removed from the endocarp, chopped, and dried in a hot air oven for 12 h at 45°C. The dried peels were ground and stored in a refrigerator for further analysis (Brahmi et al. 2021).

### 2.3 Extraction of Essential Oils

The EO was extracted following Roubi et al. (2024), through hydro‐distillation. The peels were placed in a flask with distilled water, and boiling chips were added. The flask was then heated gradually for 2 h. The organic phase (EO) was separated from the extract layers, dehydrated with anhydrous sodium sulfate, and stored for future use.

### 2.4 Characterization of Essential Oil

#### 2.4.1 Gas Chromatography–Mass Spectrometry

Gas chromatography–mass spectrometry analysis was conducted using an Agilent 7890 gas chromatograph fitted with a 5975C mass spectrometer. Aromatic compounds in EOs were identified using an Agilent Technologies HP‐5 ms column. The injector temperature was set at 150°C, and the detector temperature was 250°C. The initial temperature was 100°C for 4 min, then gradually raised to 130°C at a rate of 5°C per minute for 20 min. The ion source temperature was 230°C with a 30:1 split ratio. Helium was used as the carrier gas at a flow rate of 1.2 mL per minute. The mass analyzer scanned a range of 30–550 amu for 1 s. Diluted samples at a concentration of 10 mg/mL in methylene dichloride were injected at 1.0 μL for analysis. Bioactive components were identified by comparing mass spectra with standard spectra from NIST05.LIB. Retention indices on an HP‐5‐ms column were calculated and validated (Yang and Jin 2024).

### 2.5 Synthesis of Selenium Nanoparticles From EO

EO‐mediated selenium nanoparticles were synthesized by adding 0.5 mL of EO to 19 mL of 10 mM Na_2_SeO_3_ solutions (pH 7.4). The reaction mixture was incubated at 37°C on a rotary shaker at 120 rpm. The color change from pale yellow to ruby red indicated the reduction of selenium ions. The unreacted EO was removed by centrifuging the mixture at 6000 rpm for 11 min. The supernatant was removed, and pellets (red) were collected, washed thrice using ethanol, and dried overnight at room temperature. The dried SeNPs were stored in amber jars for further analysis (Cittrarasu et al. 2021; Moosavy et al. 2023).

### 2.6 Characterization of Selenium Nanoparticles

#### 2.6.1 Localized Surface Plasmon Resonance of SeNPs by UV–Visible Spectroscopy

The localized surface plasmon resonance (SPR) of SeNPs was determined using a Varian Cary 100 UV–visible spectrophotometer. The scanning range was set between 200 and 800‐nm wavelength. The SeNPs were dispersed in deionized water and analyzed (Tabibi et al. 2023).

#### 2.6.2 Fourier Transform Infrared Spectroscopic Analysis of SeNPs

Functional groups of EOs and SeNPs were identified using a Shimadzu FTIR (ITPrestige‐21) coupled with a horizontal attenuated total reflectance (HATR) accessory. The tested sample was dropped onto HATR plates. The wavelength of FTIR spectra ranged from 4000 to 500 cm^−1^. The relative humidity and temperature were maintained at 30% and 24°C, respectively. Approximately 200 mg of dry KBr powder tablets were molded into two transparent blank KBr tablets of 1‐mm thickness and 5‐mm diameter. Thin liquid films were prepared by adding 2 μL of tested sample to KBr tablets for infrared spectroscopy analysis. Each measurement consisted of five recordings, with four scans and 20 spectra each. Baseline correction was applied, and the curves were smoothed using OMNIC 8.0 Software. The spectral data were then imported into Unscrambler 9.7 software to standardize the normal variations (Tabibi et al. 2023).

#### 2.6.3 X‐Ray Diffraction

The X‐ray diffractometer (Ultima IV Rigaku, Japan) was utilized to determine the crystalline nature of SeNPs. The instrument was set at 40 kV with CuKα radiation at 1.22 Å. The scanning rate was set at 4° per minute (Tabibi et al. 2023). The crystallite domain size of the synthesized SeNPs was calculated using the Debye–Scherrer equation

(1) $$D=\mathrm{K\lambda}/\left(\beta \;\cos\;\theta \right)$$

where *D* = *Kλ*/*β*, where *K* is the Scherrer constant (0.98), *λ* is the wavelength (1.54), and *β* is the full width at half‐maximum (FWHM).

### 2.7 Bioactivities of Essential Oil and SeNPs

To optimize the dosage of SeNPs, various concentrations (1, 3, 5, 7, 10, 20, and 30 ppm) of the SeNPs, salt, and EO were tested for their total antioxidant content, DPPH free radical scavenging percentage, antimicrobial potential, toxicity analysis through human red blood cells membrane stabilization assay, protein denaturation assay, and in vitro anti‐pesticidal activity. The results were compared with respective standards.

#### 2.7.1 DPPH (2,2‐Diphenyl‐1‐Picrylhydrazyl) Assay

The free radical scavenging potential of EO and SeNPs was determined using the diphenyl‐picrylhydrazyl (DPPH) assay (Shandhi 2024). A 0.1 M DPPH solution was prepared by adding 3.94 g of DPPH to 50 mL of methanol. The solution was stirred well, and the total volume was adjusted to 100 mL by adding methanol. To determine the free radical scavenging potential of the tested samples, 3 mL of 0.1 M DPPH was added to 1 mL of the tested samples. The reaction mixture was incubated in the dark for 30 min. The absorbance was measured at 517 nm. Alpha‐tocopherol was used as a standard, and DPPH in methanol solution was used as the negative control. The free radical scavenging percentage was determined using the formula:

(2) $$\text{DPPH scavenging}\;\left(\%\right)=\left(\mathrm{Abs}\;\text{control}-\mathrm{Abs}\;\text{sample}\right)/\mathrm{Abs}\;\text{control}\times 100$$

where, Abs control represents the absorbance of the negative control and Abs sample represents the absorbance of the tested sample of alpha‐tocopherol, EO, and SeNPs.

#### 2.7.2 Antimicrobial Profile of EO and SeNPs

The antimicrobial potential of EO and SeNPs against tomato‐infecting strains was evaluated through a disc diffusion assay. The antimicrobial activity of EO and SeNPs was tested using microbial strains obtained from the First Fungal Culture Bank Institute of Agricultural Sciences (FFCB‐IAGS) at the University of the Punjab, Lahore. Two bacterial strains ( *Pseudomonas syringae* CP005969, *Xanthomonas campestris* CP011946) and fungal strains (*Aspergillus niger* AJ223852 and *Fusarium oxysporum* SAMN15791673) were utilized in the study. The microbial strains were cultured following the methodology described by Igoche et al. (2022). A mixture of 9 g of malt extract and 7.5 g of agar was dissolved in 250 mL of distilled water. The solution was stirred, and the final volume was adjusted to 500 mL before being sterilized by autoclaving at 121°C and 15 psi pressure. Subsequently, 15 mL of the media was poured into sterilized Petri plates and allowed to solidify at room temperature. The Petri plates were then inoculated with microbial strains, and wells were created using a cork borer. The samples were added to the wells, and the plates were incubated at 37°C for 24 h for bacterial strains and at 33°C for 5–7 days for fungal strains. The diameter of the clear zone of inhibition around the well was measured using a Vernier caliper.

#### 2.7.3 Toxicity Analysis

##### 2.7.3.1 Preparation of Human Red Blood Cell (HRBC) Suspension

The study on the stabilization activity of human red blood cell membranes was conducted following the method described by Pandey (2024). The ethics committee of the Department of Botany at Lahore College for Women University, Lahore, approved the study. Blood samples were obtained from healthy volunteers, and 0.85% NaCl was added to whole blood in a 1:1 ratio. The saline‐blood mixture was centrifuged at 3000 rpm for 10 min. The resulting pellets were washed three times with 0.85% saline solution (pH 7.2). A 10% erythrocyte solution was prepared using an isosaline solution (0.85% NaCl).

##### 2.7.3.2 Protein Denaturation Assay

The protein denaturation assay was conducted following the standard protocol outlined by Alesio and Bothun (2024). The reaction mixture was prepared by combining 50 μL of BSA with 1 mL of PBS in the tested samples across a range of concentrations. Denaturation was initiated by incubating the solution for 15 min at 70°C, with sodium diclofenac serving as the standard. Absorbance was measured at a wavelength of 660 nm, with distilled water used as the negative control. The percentage of protein denaturation activity was calculated using the formula:

(3) $$\text{Protein Denaturation}\;\left(\%\right)=\left[\left(\mathrm{Abs}\;\text{control}-\mathrm{Abs}\;\text{sample}\right)/\left(\mathrm{Abs}\;\text{control}\right)\right]\times 100$$

##### 2.7.3.3 Heat‐Induced Hemolysis

For the heat‐induced hemolysis assay, the reaction mixture was prepared by adding 0.5 mL of the tested sample or standard (sodium diclofenac) to 2 mL of hypo‐saline (0.36%) solution, 1 mL of phosphate buffer, and 0.5 mL of HRBC suspension. The mixture was then incubated for 30 min at 37°C. Following incubation, the solution was centrifuged at 3000 rpm for 10 min, and hemolysis was assessed at 560 nm. A negative control using 0.1% Triton X was included (Pandey et al. 2023). The percentage of hemolysis inhibition was determined using the formula:

(4) $$\text{Hemolysis inhibition}\;\left(\%\right)=\left(\mathrm{Abs}\;\text{sample}/\mathrm{Abs}\;\text{Control}\right)\times 100$$

#### 2.7.4 Biopesticidal Activity of SeNPs

*Tuta absoluta* (tomato pinworms) were collected from the Entomology laboratory at Ayub Agriculture Research Institute in Faisalabad. The strains were maintained at 27°C ± 2°C, 65% relative humidity, and a photoperiod of 14:10 h:D (Sangeeta et al. 2024; Alesio and Bothun 2024). The pinworms were kept on petri plates with tomato leaves treated with various solutions, and the results were compared with an untreated control and a commercial pesticide (Flubendiamide). The trays were placed in a room with a consistent temperature, and the pinworms were allowed to feed on the leaves. The feeding behavior was monitored regularly for 3 days at 24‐h intervals (24, 48, and 72 h). Subsequently, the mortality rate was calculated using the following formula:

(5) $$\text{Mortality}\;\left(\%\right)=\mathrm{No}.\;\text{of dead larvae}/\text{initial number of larvae}\times 100$$

### 2.8 Germination Assay

Based on the biological activities, the treatments that showed the best results were tested for tomato germination parameters. The seed germination assay consisted of five petri plate experiments in a completely randomized block design (CRBD) with four replications. The experiment included one tomato variety (NADIR) and 9 seed priming methods (a: 10 ppm SeNPs; b: 20 ppm SeNPs; c: 30 ppm SeNPs; d: 10 ppm Na_2_SeO_3_; e: 20 ppm Na_2_SeO_3_; f: 30 ppm Na_2_SeO_3_; g: 10 ppm EO; h: 20 ppm EO; i: 30 ppm EO; and j: Control untreated). Rubber septa were inserted into the lid of sterilized plastic petri plates (Shakar et al. 2016).

#### 2.8.1 Germination Medium

Tomato seeds (NADIR) were disinfected with 70% ethanol and washed with distilled water three times. The specified amount of each sample was spread on round filter papers at the bottom of each Petri plate. Ten surface‐sterilized seeds were then placed on the filter paper above the treatment and covered with another layer of filter paper (Whatman No. 42) to prevent seed displacement. The Petri plates were sealed with paraffin wax. Approximately 7 mL of distilled water was injected into each Petri plate through rubber septa, incubated at ±25°C with a 14‐h photoperiod and 14‐h dark period. The intensity of light was 50 μmol m^−2^ s^−1^. Radicle emergence was observed regularly at 12‐h intervals for up to 7 days, and germination parameters were noted.

The germination percentage (GP), mean germination time (MGT), days to 50% germination (T50), germination index (GI) seedling vigor index (SVI) were calculated following the methodology of Dhakal et al. (2024). The seeds with an approximate radicle length of 2 mm were considered germinated, and the total number of germinated seeds was recorded.

GP, T50, MGT, GI and SVI were calculated following the formula:

(6) $$\mathrm{GP}=\left(\mathrm{no}.\;\text{of seeds germinated}/\text{total}\;\mathrm{no}.\;\text{of seeds used}\right)\times 100$$

(7) $$T50=\mathrm{ti}+\left(\left(n/2-\mathrm{ni}\right)\left(\mathrm{tj}-\mathrm{ti}\right)\right)/\left(\mathrm{nj}-\mathrm{ni}\right)$$

where *N* is the total number of germinated seeds and *ni*, *nj* are cumulative numbers of seeds germinated by adjacent counts at time *ti* and *tj* (days), respectively; when *ni* < *N*/2 < *nj*.

(8) $$\mathrm{MGT}=\left(\sum \mathrm{Dn}\right)/\left(\sum n\right)$$

where *n* is the number of seeds germinated on day *D* and *D* is the number of days counted from the beginning of germination.

(9) $$\mathrm{GI}=\left(\text{Number of germinated seed}/\mathrm{day}\;\text{of first count}\right)+\ldots +\left(\text{number of germinated seeds}/\mathrm{day}\;\text{of final count}\right)$$

The SVI was calculated after a 14‐day seed germination period. Approximately 10 seedlings from each treatment and replicate were randomly selected, and root and shoot length and weight were measured. The roots and shoots were then oven‐dried at 72°C for 72 h, and the dry weight was recorded. The SVI was calculated using the formula provided by Dhakal et al. (2024).

(10) $$\text{Seedling Vigor Index}\;\left(\mathrm{SVI}\right)=\text{Germination}\;\left(\%\right)\times \text{Mean seedling length}\;\left(\mathrm{cm}\right)$$

(11) $$\text{Seedling Vigor Index}\;\left(\mathrm{SVI}\right)=\text{Germination}\;\left(\%\right)\times \text{Seedling}\;\mathrm{dry}\;\text{weight}\;\left(g\right)$$

### 2.9 Chlorophyll Content Estimation

Chlorophyll content of seedlings leaf samples (cotyledonary leaves) was determined. For this purpose, 300 mg of cotyledonary leaf samples were immersed in 95% ethanol for 5 min. Later on, the leaves were cut into small pieces and extracted in 80% acetone. The samples were incubated for 30 min in the dark. After the incubation period, the samples were centrifuged at 2750 rpm at 4°C for 15 min. The supernatant was stored in amber glass jars for further analysis. The chlorophyll content was measured at 645, 652, and 663 nm using an Ultraviolet–visible (UV–Vis) spectrophotometer (UV‐1800; Shimadzu 4650; Shimadzu Corporation, Kyoto, Japan) (Parveen et al. 2023).

The chlorophyll a, chlorophyll b, and total chlorophyll contents were determined using the provided equations:

(12) $${\mathrm{Chl}}^{“}a^{”}\;\mathrm{\mu g}/g\;\mathrm{FW}=\left[12.7\times A_{663}-2.69\times A_{645}\right]V/\left(1000\times W\right)$$

(13) $${\mathrm{Chl}}^{“}b^{”}\;\mathrm{\mu g}/g\;\mathrm{FW}=\left(\left[22.9\times A_{645}-4.68\times A_{663}\right]V\right)/\left(1000\times W\right)$$

(14) $$\text{Total}\;\mathrm{Chl}\;\mathrm{\mu g}/g\;\mathrm{FW}=\left(A_{652}\times 1000\times V\right)/\left(W\times 1000\right)$$

where *V* is the volume of the prepared solution and *W* is the weight of the sample used.

### 2.10 Chlorophyll Stability Index

The light transmission (%) in cotyledonary leaf samples treated with various concentrations of SeNPs, sodium selenite, and EOs was determined through the chlorophyll stability index (CSI) assay. 0.25 g of cotyledonary leaf was added to two separate test tubes, each containing 10 mL of distilled water. One test tube was heated to 55°C for 60 min, while the other was kept at room temperature as a control. The absorbance at 652 nm was measured using a UV–visible spectrophotometer for both test tubes. This protocol was followed for each treatment at different concentrations (Parveen et al. 2023). The chlorophyll stability index was calculated using the formula:

(15) $$\mathrm{CSI}\%=\left(\mathrm{OD}\;\mathrm{at}\;652\;\mathrm{nm}\;\text{of treated sample}\right)/\left(\mathrm{OD}\;\mathrm{at}\;652\;\mathrm{nm}\;\text{of control}\right)\times 100$$

### 2.11 Membrane Stability Index

The membrane stability index (MSI) was calculated using the standardized protocol outlined by Parveen et al. (2023) For this, 0.1 g of cotyledonary leaf sample was added to 10 mL of distilled water. The sample was then heated at 40°C for 30 min, and the electrical conductivity (EC1) was measured using a high‐quality conductivity meter (HM Digital AP‐2) (HM Digital India Pvt. Ltd., Alwar, India). Subsequently, the sample solution was heated at 100°C for 10 min using a water bath, and its conductivity (EC2) was measured.

(16) $$\mathrm{MSI}\%=\left[\left(\text{EC1}-\mathrm{EC}2\right)/\text{EC1}\right]\times 100$$

### 2.12 Root Ion Leakage

The root ion leakage of seedlings was evaluated by first placing 0.3 g of root samples in two separate test tubes containing 10 mL of distilled water (Parveen et al. 2023). The test tubes were then incubated for 5 min at 25°C, and the electrical conductivity was measured (EC0). Subsequently, the test tubes were incubated for an additional 12 h, and the electrical conductivity (EC1) was measured again. The test tubes were then heated in a water bath at 100°C for 30 min. After the solution cooled to room temperature, the electrical conductivity (EC2) was measured. The root ion leakage was calculated using the formula:

(17) $$\text{Relative conductivity}\;\left(\mathrm{RC}\right)=\left[\left(\text{EC1}-\mathrm{EC}0\right)/\text{EC2}\right]\times 100$$

### 2.13 Statistical Analysis

The data was summarized as mean and standard deviation (SD). Tukey's *t*‐test and Pearson's correlation test were conducted using Origin Pro 2021 integrated with Python. Tukey's post hoc test was used to compare all pairs of means.

## 3 Results

The study focused on the green synthesis of selenium nanoparticles by reducing sodium selenite with citrus fruit peel EO. Additionally, the research optimized the dosage of selenium nanoparticles for use as a nanofertilizer for tomato plants through various in vitro analyses, including DPPH free radical scavenging analysis, human red blood cell stabilization assays (protein denaturation assay and hemolysis inhibition (%)), and larvicidal activity tests.

### 3.1 Characterization of Functional Compounds Present in Citrus Fruit Peels Essential Oil

**FIGURE 1:**
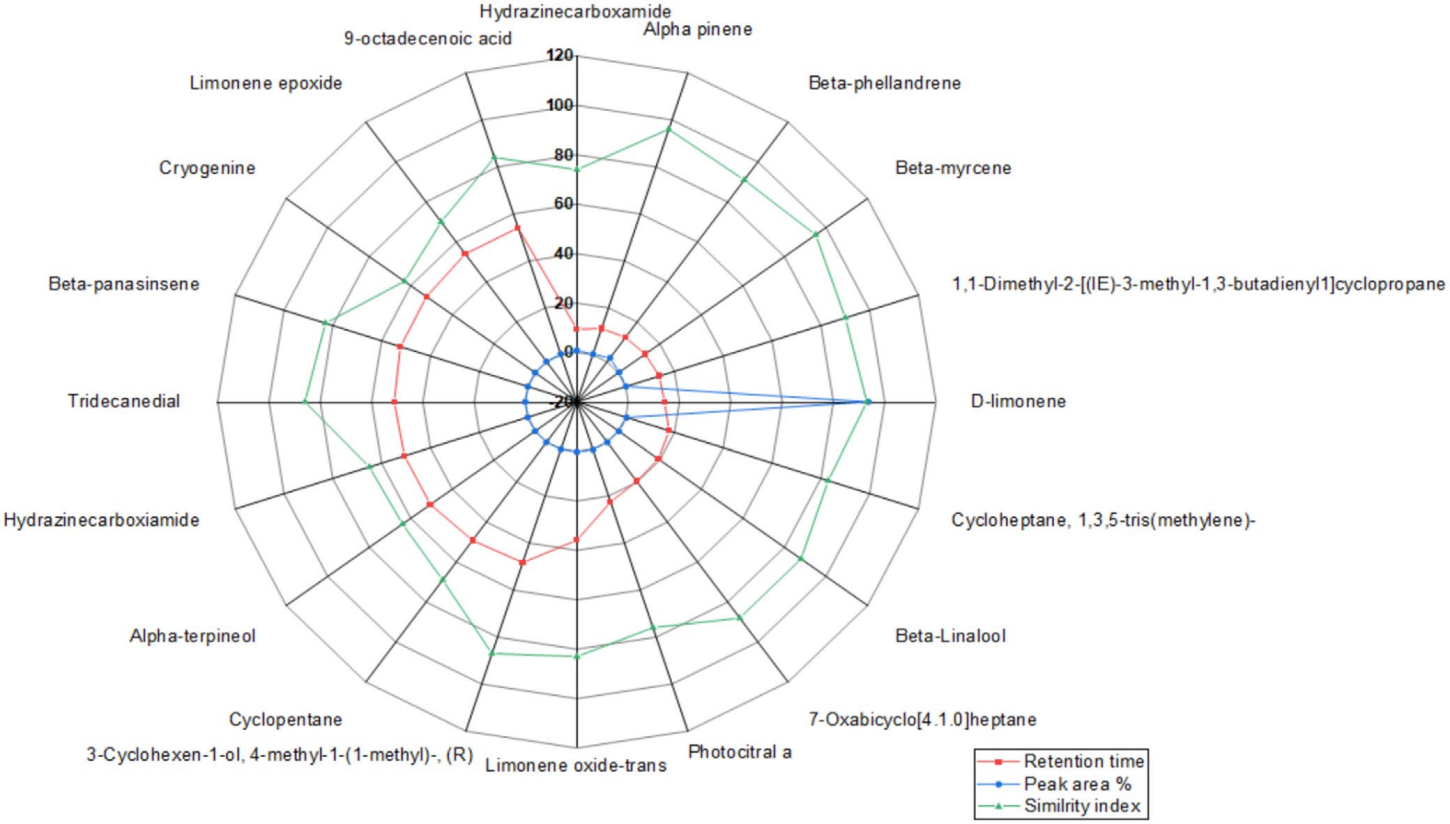
Radar plot illustrating the volatile compounds present in the citrus fruit peel essential oils. For each compound, the red line indicates retention time, the blue line represents peak area percentage, and the light blue line shows the similarity index. The radial axes show the magnitude of each parameter.

The percentage yield of EO extracted through the hydro‐distillation method was 0.37%. The functional compounds present in EOs were identified using GC–MS (Gas chromatography–mass spectrometry) and FTIR (Fourier Transform Infrared Spectrometry) (Figure 1).

#### 3.1.1 Gas Chromatography–Mass Spectrometry

Functional compounds were identified by using NIST (National Institute of Standards and Technology) library. GC–MS analysis revealed the presence of 20 functional compounds, including: Hydrazinecarboxamide (0.77%), Alpha‐pinene (0.32%), Beta‐phellandrene (1.97%), Beta‐myrcene (0.39%), 1,1‐Dimethyl‐2‐[(E)‐3‐methyl‐1,3‐butadienyl]cyclopropane (0.15%), D‐limonene (93.64%), Cycloheptane, 1,3,5‐tris(methylene) (0.43%), Beta‐Linalool (0.19%), 7‐Oxabicyclo[4.1.0]heptane (0.17%), Photocitral a (0.35%), Limonene oxide‐trans (0.21%), 3‐Cyclohexen‐1‐ol, 4‐methyl‐1‐(1‐methyl)‐, (R) (0.1%), Cyclopentane (0.17%), Alpha‐terpineol (0.1%), Hydrazinecarboxamide (0.15%), Tridecanedial (0.15%), Beta‐panasinsene (0.1%), Cryogenine (0.1%), Limonene epoxide (0.16%), and 9‐octadecenoic acid (0.38%). These compounds account for 100% constituents of the EO.

### 3.2 Characterization of Essential Oil‐Mediated Selenium Nanoparticles (SeNPs)

**FIGURE 2:**
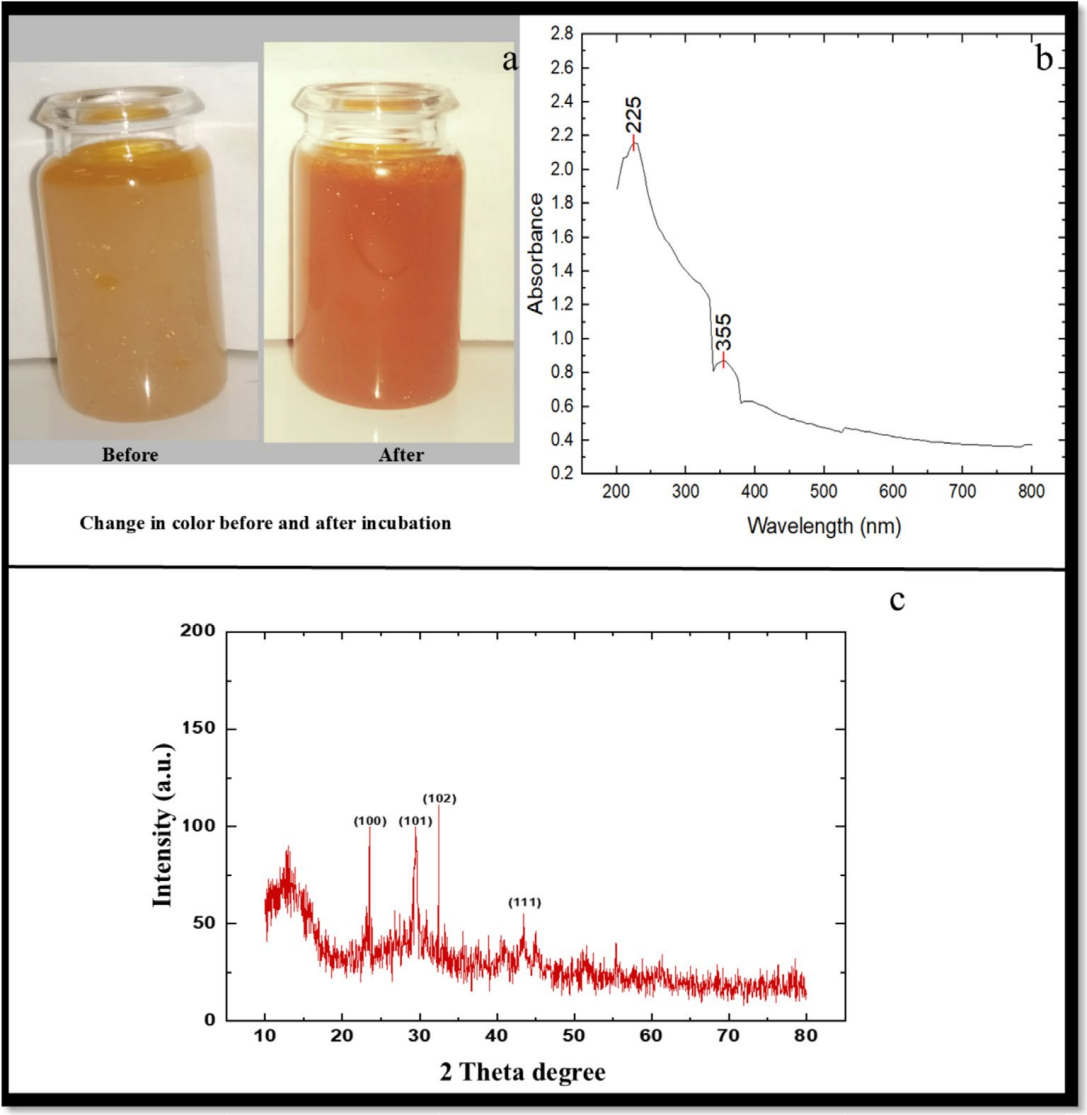
Characterization of essential oil‐mediated SeNPs: (a) change in color of reaction mixture before and after incubation; (b) UV–visible spectrum of SeNPs; and (c) XRD pattern of SeNPs.

Bioactive compounds such as amino acids, aldehydes, flavonoids, tartaric acid, NADP reductase, and secondary metabolites act as reducing agents in the synthesis of nanoparticles. When a plant extract is added to a metallic salt solution, these biological components or functional compounds facilitate the transformation of metallic ions into nanoparticles (Zhang et al. 2015). In this study, the synthesis of selenium nanoparticles using citrus fruit peel EO was confirmed by the color change from yellow to orange‐red (Figure 2a).

#### 3.2.1 Localized Surface Plasmon Resonance of SeNPs by UV–Visible Spectroscopy

The SPR of the synthesized SeNPs was confirmed through UV–visible spectra (Figure 2b). The UV –visible spectra displayed a broad peak at 265 nm, indicating the formation of SeNPs. The broadness of the peak suggested the polydispersive nature of SeNPs.

#### 3.2.2 X‐Ray Diffraction Analysis

X‐ray analysis confirmed the crystalline nature of the selenium nanoparticles synthesized using EO. The XRD diffraction pattern exhibited peaks at 23.6°, 29.6°, 32°, and 43.6°, which were ascribed to 100, 101, 102, and 111, consistent with the JCPDS 06–0362 standard. The broadening of the peaks indicated the nanoscale dimensions of the SeNPs. According to the Debye–Scherrer equation, the average crystalline nanoparticle size was determined to be 6.4 nm (Figure 2c).

#### 3.2.3 FTIR Analysis

**FIGURE 3:**
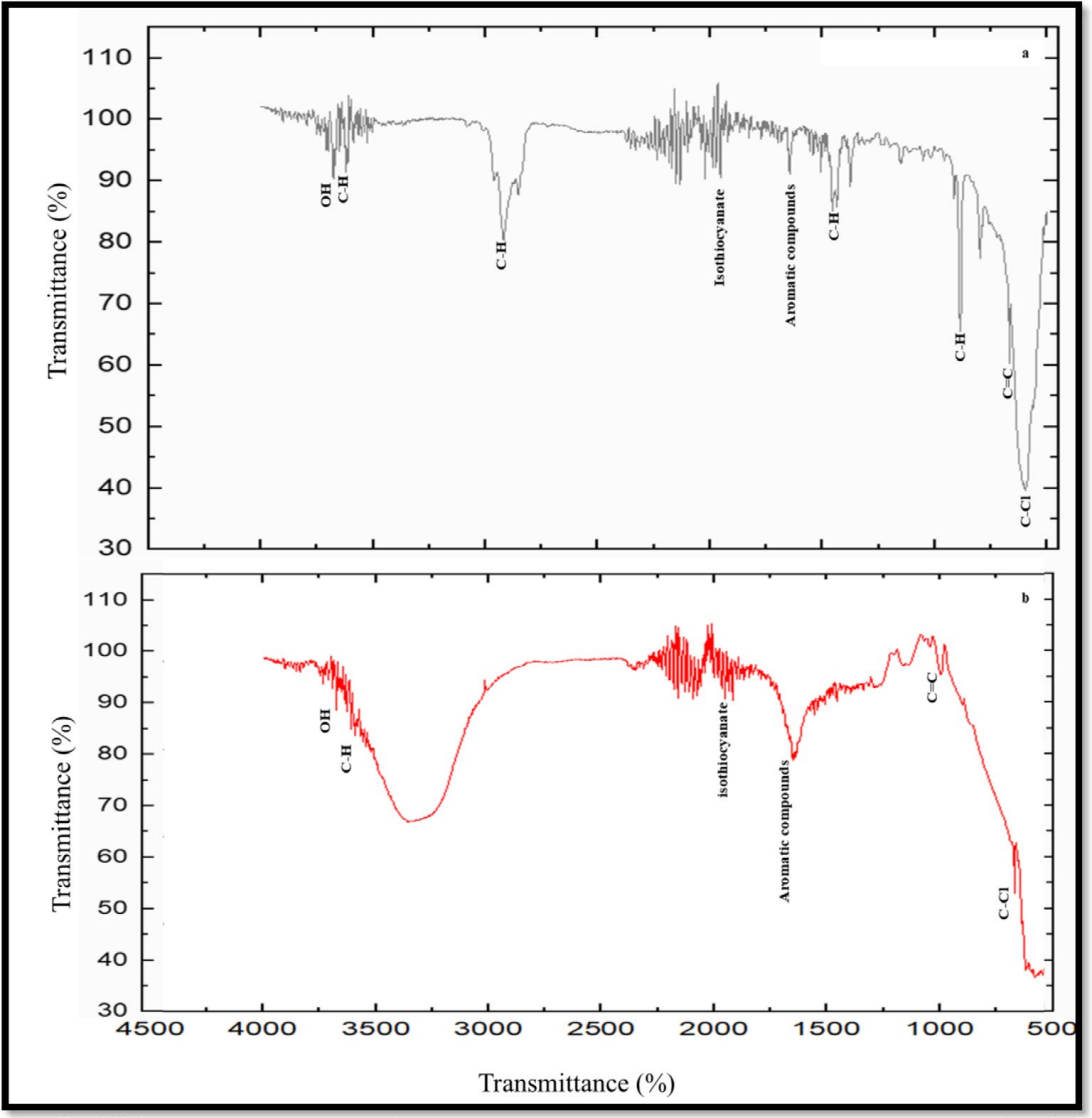
FTIR spectra of (a) essential oil (EO) and (b) essential oil‐mediated selenium nanoparticles (SeNPs).

The FTIR spectrum identified the functional groups of bioactive compounds based on their peak values in the infrared region, confirming the presence of alkanes (2915.6 cm^−1^), alkenes (3622.4 cm^−1^), amines (2853.6 cm^−1^), alkyl halides (3660 cm^−1^), aromatic compounds (1953 cm^−1^), isothiocyanate (2015.5 cm^−1^), alcohols (594.97 cm^−1^), and carboxylic acids (1636.11 cm^−1^) in the EO (Figure 3a). The FTIR analysis also revealed the potential functional groups of bioactive compounds on the surface of selenium nanoparticles (Figure 3b). A strong peak at 663.5 cm^−1^ was identified as the alkyl halides (C—X) stretch. The strong bands at 891 and 990.18 cm^−1^ indicated C=C bending, while the peak at 1643 cm^−1^ corresponded to C=C stretching. The absorption peak at 1941 cm^−1^ was attributed to the C—H bending of aromatic compounds. A strong peak at 2066 cm^−1^ was related to *N*=C=S stretching of isothiocyanate, and the absorption peak at 2169 cm^−1^ was attributed to S—C≡N stretching of thiocyanate. A broad peak at 3354 cm^−1^ and a sharp peak at 3669 cm^−1^ represented the OH stretching of the alcohol group. These results suggest that the bioactive compounds present in citrus fruit peels EO play a role in the formation and stabilization of selenium nanoparticles.

### 3.3 In Vitro Pharmacological Profile Tested Samples

The pharmacological profile at various concentrations (1, 3, 5, 7, 10, 20 and 30 ppm) of EO, precursor salt solution, and SeNPs was evaluated using the DPPH free radical scavenging assay, antimicrobial activity through the disc diffusion method, human red blood cell membrane stabilization assay, and larvicidal activity, which was measured as a percentage. The results were compared with commercial standards, respectively.

#### 3.3.1 DPPH Free Radical Scavenging (%)

**FIGURE 4:**
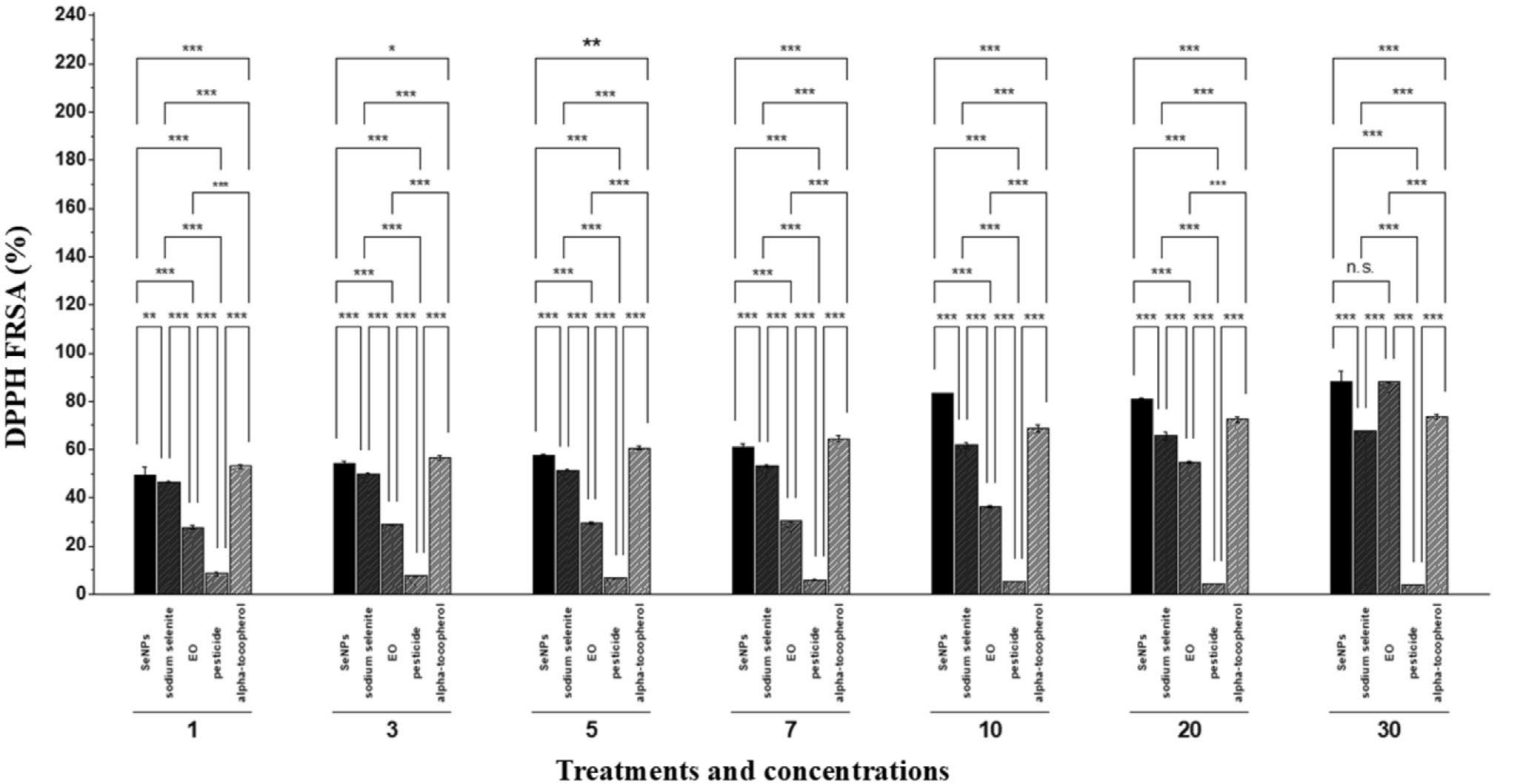
DPPH free radical scavenging profile. Selenium nanoparticles (SeNPs), essential oil (EO); pesticide (Flubendamide); standard (alpha‐tocopherol). 1, 3, 5, 7, 10, 20, and 30 are concentrations in ppm. *p < 0.05; **p < 0.01; ***p < 0.001; ns, not significant.

The DPPH free radical scavenging assay was performed to assess the antioxidant capacity of the EO, precursor salt solution, and SeNPs. A concentration‐dependent increase was observed, Figure 3 showing a consistent significant difference among mean values. SeNPs and sodium selenite exhibited a highly significant difference over an extended period. The pesticide's free radical scavenging potential was lower, indicating a negative impact. SeNPs showed the highest free radical scavenging activity (%) at a concentration of 30 ppm (88 ± 0.8), followed by 10 ppm (83.1 ± 0.3) (Figure 4). Alpha‐tocopherol had a moderate effect on the antioxidant profile compared to SeNPs.

#### 3.3.2 Antimicrobial Profile of Nanoparticles

**FIGURE 5:**
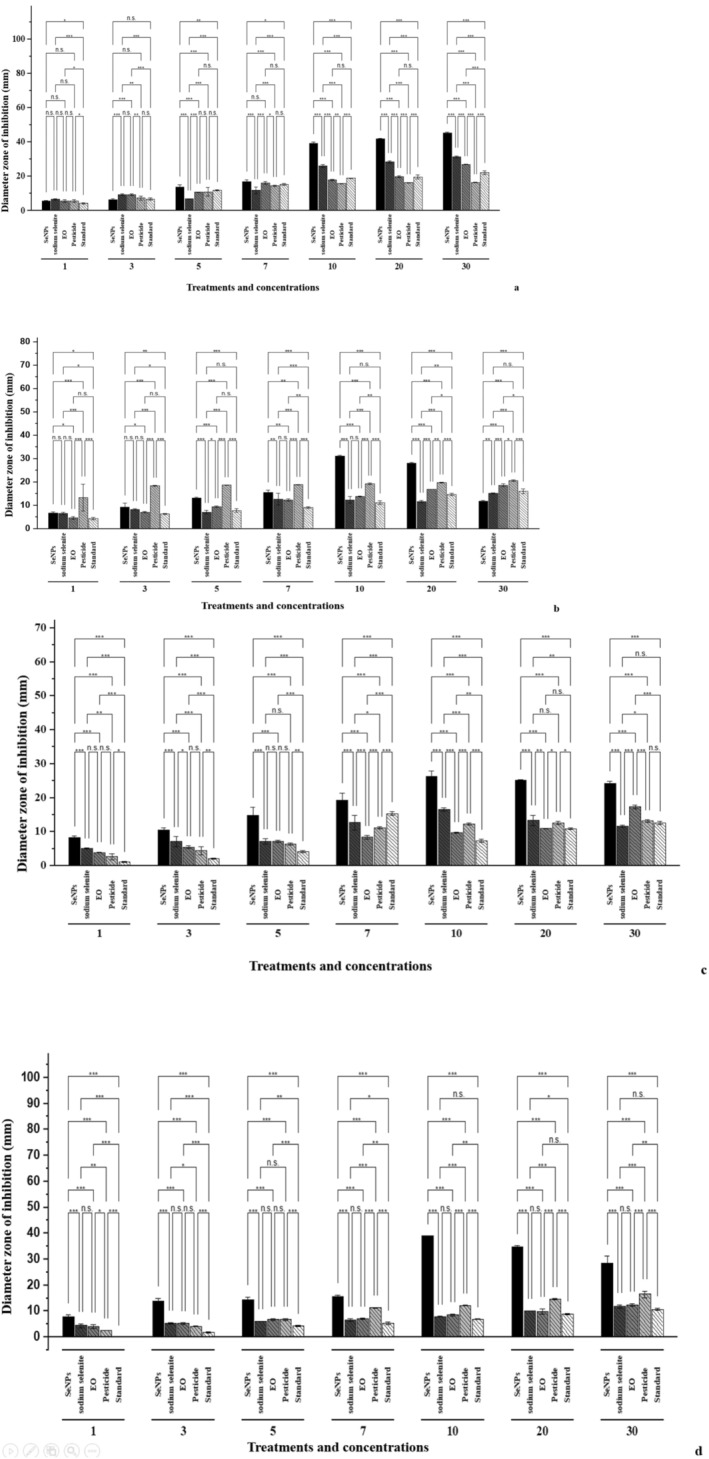
Antimicrobial profile of tested samples: (a) antibacterial potential against *P. syringae* , (b) antibacterial potential against *X. campestris* , (c) antifungal potential against *A. niger* , and (d) antifungal potential against *F. oxysporum*. Selenium nanoparticles (SeNPs), essential oil (EO); pesticide (Flubendamide); standard (amoxicillin/fluconazole). 1, 3, 5, 7, 10, 20, and 30 are concentrations in ppm. *p < 0.05; **p < 0.01; ***p < 0.001; ns, not significant.

The antimicrobial profile of green synthesized SeNPs, precursor salt, and EO was analyzed using the disc diffusion method and compared with commercially available standard antibiotics. Two bacterial strains ( *Pseudomonas aeruginosa* and *Xanthomonas campestris* ) and fungal strains (*Aspergillus niger* and *Fusarium oxysporum*) were evaluated. The results showed that increasing concentrations of SeNPs significantly enhanced the antimicrobial inhibition zones. The pesticide consistently exhibited a decrease in antimicrobial resistance across all tested concentrations, indicating a negative impact on antibacterial resistance. EOs showed a moderate increase in the zone of inhibition, while standard antibiotics (Fluconazole/Amoxicillin) showed a significant increase in resistance against the tested strains, although the values were lower than those of SeNPs (Figure 5a–d). At 10 ppm, SeNPs exhibited the highest zone of inhibition, with 39.3 and 28.1 mm against *P. syringae* and *X. campestris* , respectively. Similarly, 10 ppm SeNPs showed the highest antifungal activity, with 26.3 and 39 mm against *A. niger* and *F. oxysporum*, respectively, suggesting that higher concentrations of SeNPs enhance antifungal potency.

#### 3.3.3 Hemolysis Inhibition (%)

**FIGURE 6:**
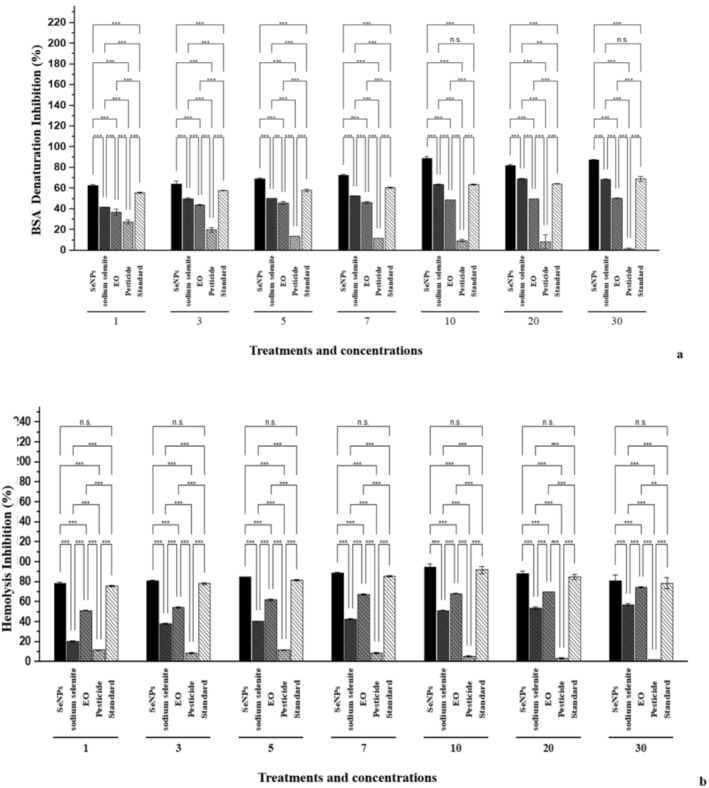
Anti‐inflammatory potential: (a) protein denaturation percentage and (b) hemolysis inhibition %. Selenium nanoparticles (SeNPs), essential oil (EO); pesticide (Flubendamide); standard (sodium diclofenac). 1, 3, 5, 7, 10, 20, and 30 are concentrations in ppm. *p < 0.05; **p < 0.01; ***p < 0.001; ns, not significant.

The anti‐inflammatory activity of the tested samples was observed through the human red blood cell membrane stabilization method and protein denaturation assay (Figure 6a,b). SeNPs at all concentrations showed the highest potential for protein denaturation and inhibition of hemolysis.

#### 3.3.4 *Tuta absoluta* Larvicidal Activity

This study assessed the effectiveness of EOs, sodium selenite, selenium nanoparticles (SeNPs), and a commercial standard pesticide on fourth instar *Tuta absoluta* larvae at varying concentrations. The results revealed that SeNPs had a more pronounced impact compared to sodium selenite against *Tuta absoluta*. The mortality percentage (MO%) of *T. absoluta* treated with 10 ppm SeNPs was 96.33 ± 0.15, followed by 20 ppm SeNPs at 87 ± 0.2 after 72 h (Table 1). The commercial pesticide (Flubendamide) showed a lower larvicidal effect at a concentration of 1 ppm, indicating a reduced or negligible biocidal effect at this concentration.

**TABLE 1:** Larvicidal effect of EO, SeNPs, and sodium selenite under different concentrations.

Treatments	Conc.	Mortality rate (%) (24 h)	Mortality rate (%) (48 h)	Mortality rate (%) (72 h)
SeNPs	1 ppm	3.1 ± 0.1*	3.72 ± 0.1*	20.67 ± 0.6***
Sodium selenite		1.07 ± 0.1*	1.29 ± 0.2*	6.86 ± 0.4***
EO		2.6 ± 0.6^ns^	3.2 ± 0.7^ns^	12.16 ± 0.26^ns^
Pesticide		0.56 ± 0.3*	0.68 ± 0.4*	2.54 ± 0.14***
SeNPs	3 ppm	4.96 ± 0.1**	5.9 ± 0.1**	36 ± 0.26***
Sodium selenite		2.36 ± 0.7**	2.84 ± 0.9**	10.7 ± 0.33***
EO		3.9 ± 0.1^ns^	4.76 ± 0.1^ns^	18.08 ± 0.3**
Pesticide		1.96 ± 0.1*	2.36 ± 0.1**	8.9 ± 0.3***
SeNPs	5 ppm	7.8 ± 0.3**	9.4 ± 0.3**	42.3 ± 0.15***
Sodium selenite		4.16 ± 0.3**	5 ± 0.3**	19 ± 0.13***
EO		5.3 ± 0.5^ns^	6.36 ± 0.6^ns^	24.1 ± 0.24^ns^
Pesticide		3.06667 ± 0.1*	3.68 ± 0.1*	13.9 ± 0.5**
SeNPs	7 ppm	13.66 ± 0.32**	16.4 ± 0.39**	45.66 ± 0.35**
Sodium selenite		5.73 ± 0.6**	6.88 ± 0.8**	26.144 ± 0.29**
EO		5.67 ± 0.6^ns^	6.8 ± 0.7**	25.84 ± 0.28^ns^
Pesticide		4.8 ± 0.3^ns^	5.8 ± 0.3^ns^	22.04 ± 0.13^ns^
SeNPs	10 ppm	56 ± 0.2***	67.2 ± 0.24***	96.33 ± 0.15***
Sodium selenite		9.2 ± 0.5***	11.12 ± 0.6***	42.6 ± 0.2***
EO		12 ± 0.1**	14.4 ± 0.12***	54.72 ± 0.46**
Pesticide		7.6 ± 0.15***	9.12 ± 0.6***	34.6 ± 0.24**
SeNPs	20 ppm	51.6 ± 0.6***	62 ± 0.7***	87 ± 0.2***
Sodium selenite		13.3 ± 0.15***	16 ± 0.18***	60.8 ± 0.7***
EO		12.3 ± 0.6^ns^	14.8 ± 0.7^ns^	56.24 ± 0.26^ns^
Pesticide		9.16 ± 0.3**	11 ± 0.3***	41.8 ± 0.13***
SeNPs	30 ppm	54 ± 0.1***	64.8 ± 0.12***	84.6 ± 0.45***
Sodium selenite		15.3 ± 0.1***	18.4 ± 0.25***	69.92 ± 0.9***
EO		14 ± 0.1^ns^	16.8 ± 0.12^ns^	63.84 ± 0.4*
Pesticide		11.7 ± 0.4*	14.04 ± 0.5*	53.3 ± 0.2**

Abbreviation: ns, not significant.

* *p* < 0.05.

** *p* < 0.01.

*** *p* < 0.001.

#### 3.3.5 Correlation Analysis

**FIGURE 7:**
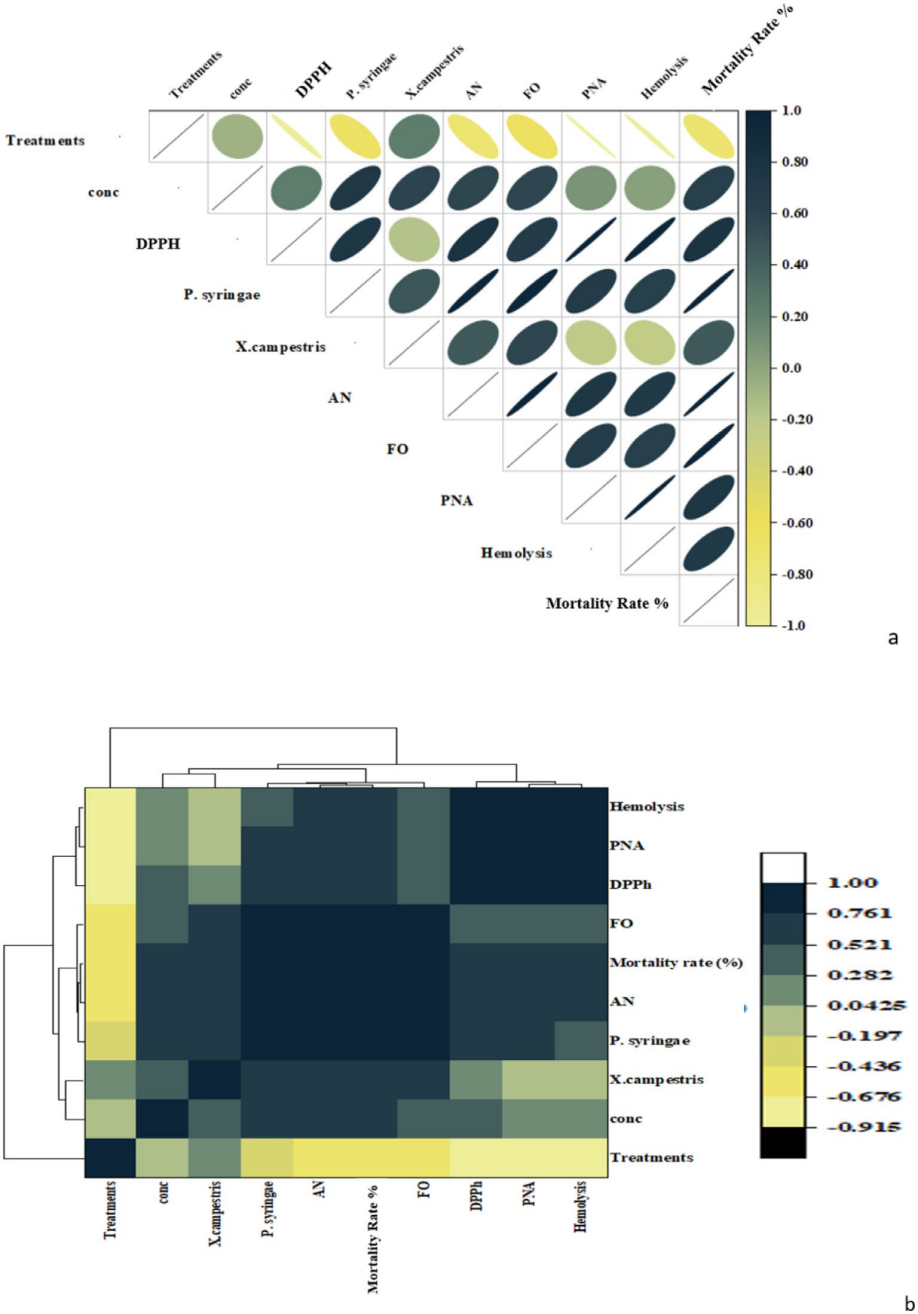
Correlation analysis among treatments and biological activities. (a) Pearson correlation analysis and (b) Heatmap with dendrogram analysis. The dark blue to white color indicates a strong positive correlation, while yellow to black shows negative correlations; AN, *Aspergillus niger*; conc., concentrations; DPPH, 2,2‐diphenyl‐1‐picrylhydrazyl; FO, *Fusarium oxysporum*; PNA, protein denaturation assay.

Pearson correlation analysis revealed significant positive relationships between sample concentrations and DPPH free radical scavenging percentage (*r* ^2^ = 0.35), *X. campestris* (*r* ^2^ = 0.73), and mortality rate (%) (*r* ^2^ = 0.64), indicating that higher concentrations enhance antioxidant capacity and antimicrobial activity. A strong negative correlation was observed between treatments and hemolysis (*r* ^2^ = 0.9), suggesting that treatments reduce toxicity. The heat map with dendrogram illustrated the relationships among variables, with dark blue shades indicating strong positive associations. Hemolysis showed a strong positive correlation with protein denaturation inhibition (%) and *Fusarium oxysporum*, suggesting that higher hemolysis inhibition is due to strong antioxidant properties, protein denaturation inhibition, and susceptibility to *Fusarium oxysporum*. Protein denaturation inhibition (%) exhibited a strong association with DPPH and *Fusarium oxysporum* activity. Yellow to black shades in the heat map indicated an inverse relationship among variables. *F. oxysporum* was found to have a strong negative correlation with hemolysis, indicating that an increase in antimicrobial potential leads to a decrease in toxicity (Figure 7).

### 3.4 Germination Parameters

The treatments that showed significant biological effects were tested to assess the influence of nanoselenium on tomato seeds. Different germination parameters such as germination percentage, 50% germination time (T50), Mean germination time, GI, and SVI were measured. Biochemical characteristics like chlorophyll content, chlorophyll content stability index, MSI, and root ion leakage were also assessed.

#### 3.4.1 Germination Parameters

**FIGURE 8:**
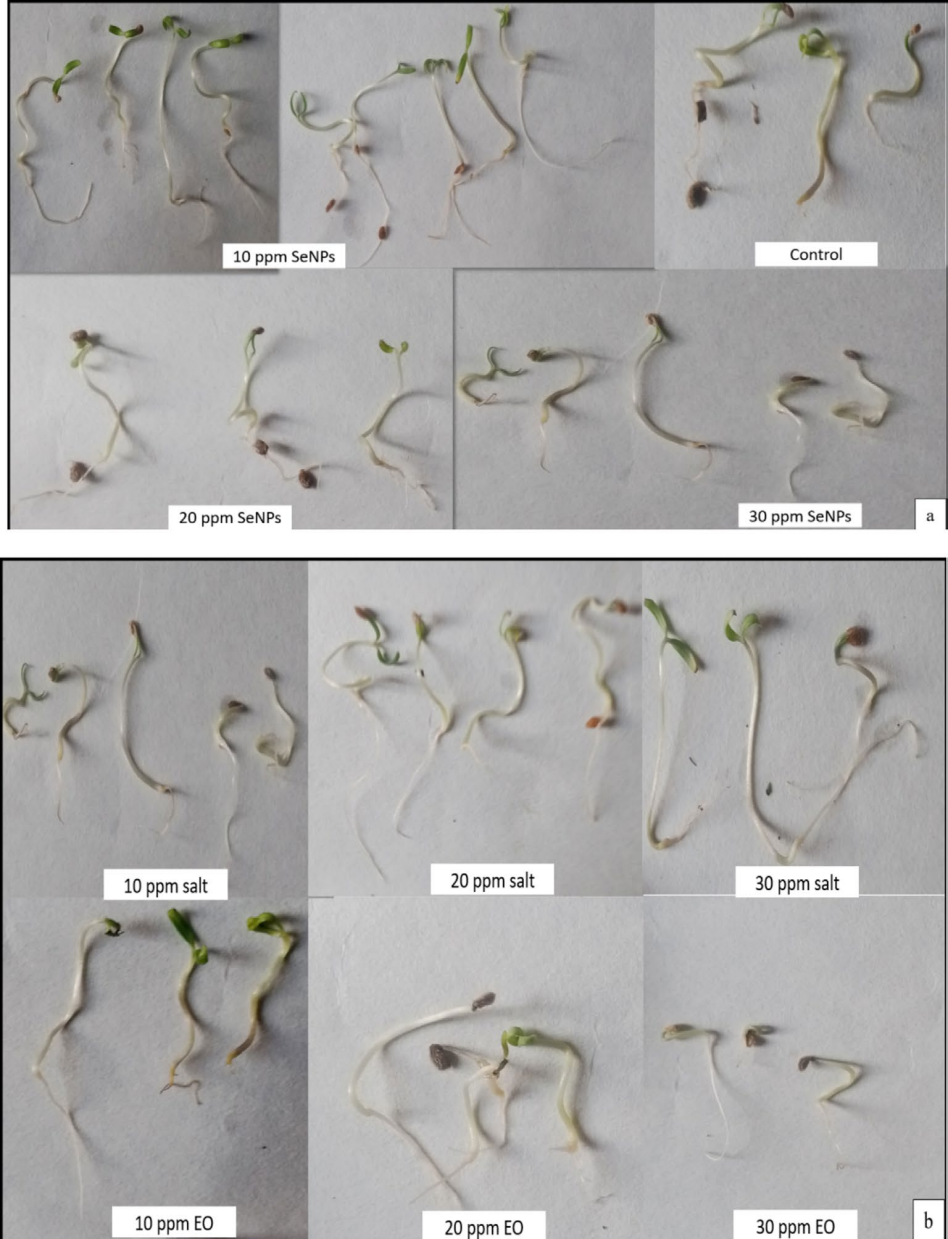
Seedlings of tomato from seed priming with EO, sodium selenite, and SeNPs: (a) nanoprimed seedlings; (b) sodium selenite and EO primed seed.

**FIGURE 9:**
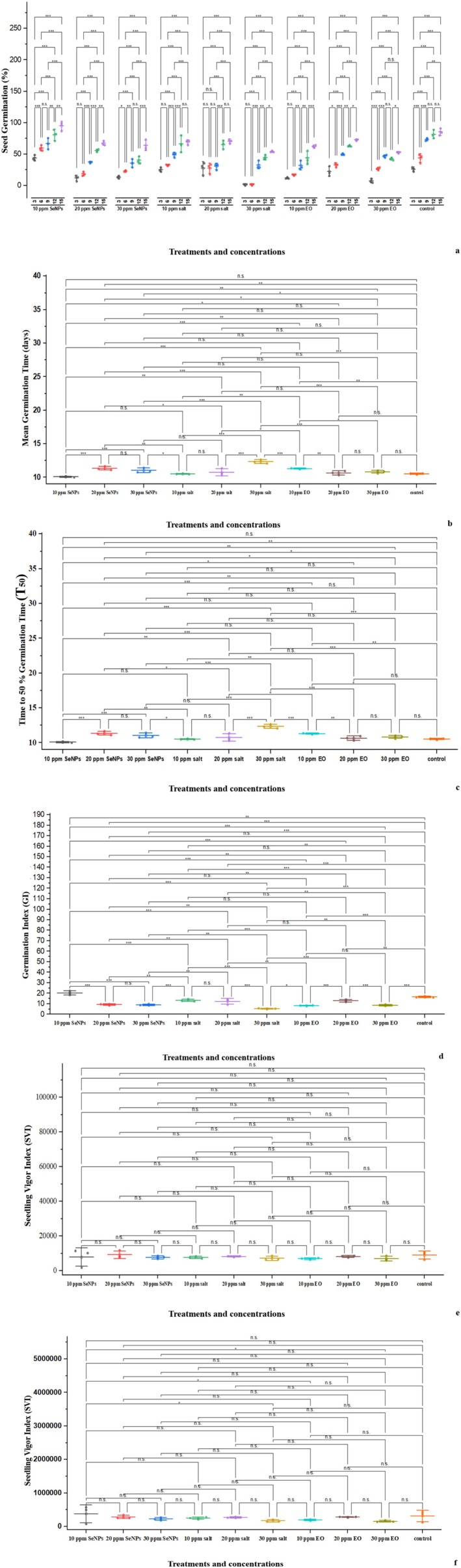
Effect of different treatments and concentrations on seed germination parameters of tomato seeds: (a) germination percentage, (b) time required for 50% seeds to germinate, (c) mean germination time, (d) germination index, (e) seedling vigor index (SVI), and (f) seedling vigor index. Close or tight clustering in data points indicates that treatments had more consistent effect on tested parameter with less variation among triplicates data whereas, wide spread clustering indicates variations among replication highlighting inconsistency. *p < 0.05; **p < 0.01; ***p < 0.001; ns, not significant.

The germination percentage indicates the number of seeds sprouted over different time intervals. Figure 8 illustrates that all treatments enhance seedling growth and physiological characteristics at the optimal concentration, but higher concentrations may sometimes delay or hinder germination. There was a significant difference among the mean values of the treatments. The highest seed germination percentage, 94.81%, was observed in 10 ppm of SeNPs, followed by 85.18% in the control on day 15 and 80.7% in 10 ppm SeNPs on day 12 (Figure 9a). The time taken for 50% of the seeds to germinate for each treatment is shown in Figure 9b. SeNPs had the lowest T50 value compared to other treatments. The 10 ppm SeNPs exhibited significantly lower T50 values compared to other treatments and the control. The average time for seeds to germinate was also evaluated for each treatment individually and presented in Figure 9c. The 10 ppm SeNPs showed a lower mean germination value, indicating the effectiveness of the treatment in speeding up the germination process. The GI was also calculated, indicating the best treatment with significant germination performance. The GI combines germination percentage and speed, indicating overall germination performance. The maximum GI was observed with 10 ppm SeNPs (20.2 ± 1.9). High SVI‐I and SVI‐II were observed with 10 ppm SeNPs, indicating strong and healthy growth of seedlings.

#### 3.4.2 Photosynthetic Pigment Content

The impact of different treatments on photosynthetic pigments was examined, revealing that SeNPs and EO treatments led to significant increases in chlorophyll a, b, and total chlorophyll content compared to the control, sodium selenite, and EO treated seedlings. The 10 ppm SeNPs seedlings were observed to have higher chlorophyll a, chlorophyll b, and total chlorophyll content (266.3 ± 1.5, 424.1 ± 1.03 and 1008.3 ± 13.5), respectively. This suggests that SeNPs had a notable effect on the synthesis of photosynthetic pigments. Additionally, SeNPs exhibited a significant chlorophyll stability index of 84%, higher than the untreated control's 67.6%. The MSI of SeNPs treated seedlings was also higher, indicating effective cell protection. Furthermore, the root ion leakage (RIL) of SeNPs treated seedlings was lower than that of Salt, EO, and the untreated control, suggesting healthier root health (Table 2).

**TABLE 2:** Chlorophyll content, membrane stabilization index and root ion leakage of seedlings treated with different concentrations of EO, SeNPs, and sodium selenite.

Treatments	Chl a (μg/g fresh W)	Chl b (μg/g fresh W)	Total Chl (μg/g fresh W)	CSI (%)	MSI (%)	RIL (%)
SeNPs‐10 ppm	266 ± 0.15***	424.1 ± 1***	1008 ± 0.4^ns^	84.6 ± 2.6***	94 ± 1***	0.18 ± 0.01***
SeNPs‐20 ppm	222 ± 1***	410 ± 1.5***	992.6 ± 2.5^ns^	65.1 ± 0.17***	91 ± 0.5**	0.9 ± 0.01***
SeNPs 30 ppm	244.6 ± 1.5***	406 ± 0.5^ns^	945.3 ± 0.5***	63.2 ± 0.72***	86 ± 0.5^ns^	1.8 ± 0.05***
Salt‐10 ppm	52.6 ± 1.03***	90 ± 1	275 ± 0.45***	32.4 ± 0.5***	79 ± 0.5^ns^	9.26 ± 0.4**
Salt‐20 ppm	54.8 ± 0*	112 ± 1.1***	281.6 ± 0.7^ns^	54.8 ± 0.23***	82 ± 1^ns^	9.9 ± 0.08***
Salt‐30 ppm	66.7 ± 0.5***	156 ± 3.7***	346.6 ± 2.08***	22.3 ± 1.2***	27 ± 1^ns^	6.8 ± 0.13***
EO‐10 ppm	95.3 ± 0.5***	262 ± 3.4***	485 ± 1***	19 ± 1^ns^	81.5 ± 0.5^ns^	4.41 ± 0.3***
EO‐20 ppm	157 ± 2.1***	267 ± 1***	824.3 ± 2.08***	18.8 ± 0.2^ns^	76.6 ± 0.5^ns^	4.3 ± 0.1^ns^
EO‐30‐ppm	49.8 ± 0.2***	404.6 ± 2^ns^	353.3 ± 2.8***	23.6 ± 2.1***	58.6 ± 1.2^ns^	2.4 ± 0.2 ***
Control	152 ± 1***	257 ± 0.4***	810 ± 0.10***	67.6 ± 1.5***	38 ± 0.5^ns^	6.9 ± 0.06***

Abbreviations: Chl a, chlorophyll a; Chl b, chlorophyll b; CSI, chlorophyll stability index; EO: essential oil; MSI, membrane stability index; ns, not significant; ppm, parts per million; Salt, sodium selenite; SeNPs: selenium nanoparticles; Total Chl, total chlorophyll.

* *p* < 0.05.

** *p* < 0.01.

*** *p* < 0.001.

#### 3.4.3 Correlation Analysis

**FIGURE 10:**
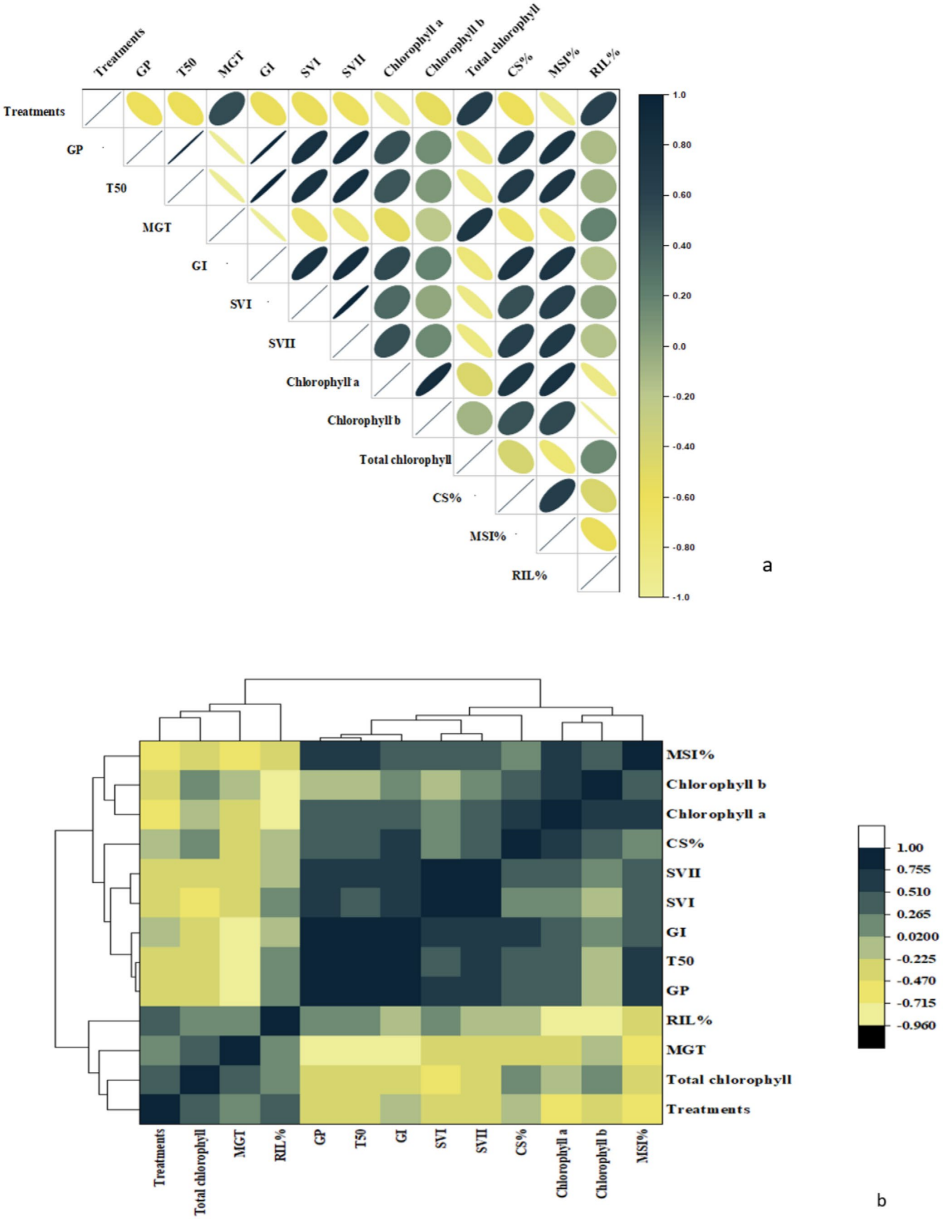
Correlation analysis among treatments and germination parameters. (a) Pearson correlation analysis, (b) heatmap with dendrogram analysis. The dark blue to white color indicates a strong positive correlation, while yellow to black shows negative correlations.

Pearson correlation analysis revealed a positive correlation between chlorophyll a, b, and total chlorophyll content. The membrane stabilization index (%) was significantly associated with chlorophyll a, b, and total chlorophyll content, suggesting that an increase in membrane integrity helps retain chlorophyll content under stress conditions (*r* ^2^ = −0.39). A heatmap with a dendrogram separated growth inhibitors from growth–promoting traits. Chlorophyll a, b, and total chlorophyll content showed a positive correlation with each other. The membrane stabilization index (%) is significantly associated with chlorophyll a, b, and total chlorophyll content, indicating that an increase in membrane integrity retains the chlorophyll content under stress conditions. The mean germination time has a negative correlation with germination percentage, GI, and SVI, indicating that faster germination is linked with a higher germination rate (%) and vigor index. Relative ion leakage (%) as an indicator of stress trait is negatively correlated with vigor chlorophyll, as higher stress conditions cause a decrease in germination rate (%) and degradation of chlorophyll (Figure 10).

## 4 Discussion

Tomato is a popular food crop from the Solanaceae family, widely consumed and used in various dishes. It is the second most cultivated crop globally and is primarily grown as an annual crop, although it can also be biennial or perennial (Sattar et al. 2024; Wang et al. 2023). However, tomatoes are delicate plants and are vulnerable to pests and microbial pathogens, with insect attacks being a major challenge in tomato production. Insects not only cause physical damage to the plants but also create openings for microbial pathogens like bacteria, viruses, and fungi to enter the plants (Wang et al. 2023). Cammarano et al. (2022) have projected a 6% decrease in global tomato production by 2050 due to factors such as extreme temperatures and the presence of pathogens like *Tuta absoluta*, black rot *Xanthomonas vesicatoria* , bacterial speck *Pseudomonas syringae* pv. tomato, and bacterial canker *Clavibacter michiganensis* subsp. *michiganensis*. Pests and microbial infections are mostly treated with synthetic chemicals, which are posing serious threats to the environment and human health (Cammarano et al. 2022). Additionally, these chemicals are developing resistance against pathogens. GC–MS analysis of citrus fruit peel EO revealed the presence of various compounds, including oxygenated monoterpenes (Alpha‐terpineol, Photocitral A, Beta‐Linalool, 3‐Cyclohexen‐1‐ol, 4‐methyl‐1‐(1‐methylethyl)‐, (R), Limonene oxide‐trans, Limonene epoxide); Monoterpenes (Beta‐phellandrene, D‐limonene, Beta‐myrcene, Beta‐panasinsene, and Alpha‐pinene); Alkyl Hydrocarbons and Derivatives (Cyclopentane, 1,1‐Dimethyl‐2‐[(1E)‐3‐methyl‐1,3‐butadienyl]cyclopropane, Cycloheptane, 1,3,5‐tris(methylene)‐(polycyclic hydrocarbon)); Carboxamides/Nitrogen‐Containing Compounds (Hydrazinecarboxamide, Cryogenine); Ethers/Heterocyclic Compounds: (7‐Oxabicyclo[4.1.0]heptane) and Fatty Acids and Aldehydes (9‐Octadecenoic acid, Tridecanedial) (Figure 1).

The FTIR analysis confirmed the presence of various functional groups in the EO‐mediated selenium nanoparticles, including alkanes, alkenes, alkyl halides, amines, aromatic compounds, isothiocyanate, alcohols and carboxylic acids. The formation of the nanoparticles was indicated by a color change from yellow to orange‐red and SPR was observed at 265 nm (Figure 2). Salem et al. (2021) observed the SPR at 275 nm of *Penicillium corylophilum* biomass mediated SeNPs. The FTIR spectra of the SeNPs exhibited absorption peaks at 663.5, 891, 990.18, 1643, 1941, 2066, 2169, 3354 and 3669 cm^−1^, confirming the involvement of alkyl halides, carboxylic acids, alcohols, aromatic compounds and isothiocyanate in the synthesis and stabilization of the nanoparticles. Salem et al. (2021) utilized *P. corylophilum* biomass filtrate to reduce sodium selenite, and the FT‐IR spectrum of the fungal biomass showed characteristic peaks (1623 and 3291 cm^−1^) corresponding to C=O, C=N, C=C, O—H and N—H groups. The fungal biomass‐mediated SeNPs also exhibited distinct peaks (3218 and 1569 cm^−1^) indicating the presence of specific functional groups. The XRD pattern showed the intense peaks at 23.6°, 29.6°, 32° and 43.6°. The results were consistent with the JCPDS No. 06‐0362 (Deng et al. 2017).

The study evaluated EOs, sodium selenite (precursor salt), and EO‐mediated SeNPs across different concentrations (1, 3, 5, 7, 10, 20 and 30 ppm) for their biological properties through DPPH free radical scavenging assay, antimicrobial potential (against tomato‐infecting pathogens), anti‐inflammatory effects (protein denaturation assay and hemolysis inhibition), and pesticidal activity (against *Tuta absoluta*) (Figure 3).

EO, SeNPs, and sodium selenite demonstrated strong DPPH free radical scavenging activity, antimicrobial, anti‐inflammatory, and pesticidal potential. In contrast, flubendamide (a commercial pesticide) exhibited weak antioxidant, antimicrobial, and anti‐inflammatory properties. Arafa et al. (2024) documented that flubendamide is a novel pesticide widely used in agriculture and can be hazardous to humans, as it can trigger inflammation, oxidative stress, and disrupt the cell cycle in human prostate epithelial cells. Their study also found that *Trichoderma aureoviride*‐mediated SeNPs can mitigate the cytotoxic effects caused by flubendamide in human epithelial cells, which supports the findings of the present study. Correlation analysis revealed that an increase in concentration causes a significant increase in DPPH free radical scavenging percentage, mortality rate (%), and antimicrobial potential against *X. campestris* , indicating that an increase in concentration leads to an increase in antioxidant, antimicrobial, and pesticidal effects. Antimicrobial activity showed a strong positive correlation with antioxidant and hemolysis inhibition percentage, suggesting that an increase in antioxidant activity results in an increase in antimicrobial activity and a decrease in cytotoxicity (Figure 6). The antimicrobial potential of EOs is due to the presence of carboxamide derivative compounds, monoterpenes, and fatty acid derivative compounds. Sharshira and Hamada (2011) documented that carboxamide derivatives exhibit strong antimicrobial potential. Kong et al. (2024) reported monoterpenes as potential antimicrobials against various infectious pathogens. Qasim et al. (2024) documented that monoterpenes possess strong antimicrobial, insecticidal, pesticidal, and antifeedant potential. Carboxamide groups are used in seed priming, foliar application, or soil treatment to control fungal growth. They are also used in combination with other fungicides or insecticides (Qasim et al. 2024; Kong et al. 2024). The bioactive compounds in EO exhibit antimicrobial properties by disrupting cell membranes, inhibiting ATP synthesis, and causing cytolytic leakage (Wongkattiya et al. 2019; Yang and Jin 2024). The significant positive correlation between pest mortality rate percentage and DPPH free radical scavenging, antibacterial, and antifungal activity indicates that pest invasion enhances the rate of attack by microbial pathogens. Therefore, an increase in the mortality rate (%) causes a decrease in pathogen infection. Nanoparticles penetrate microbial membranes and inactivate microbial cells by disrupting the integrity of cell membranes and cytoplasmic membranes, inhibiting ATP synthesis, and initiating oxidative stress through the production of reactive oxygen species (Nisar et al. 2019).

To evaluate the effectiveness of EO‐mediated SeNPs as a nano seed priming agent, tomato seeds were treated with different concentrations (10, 20, and 30 ppm) of SeNPs solution. The results were compared with hydroprimed, sodium selenite‐primed, and EO‐primed seeds. It was observed that 10 ppm SeNPs enhanced the seed germination percentage by widening the pores in the seed coat Wojtyla et al. (2024). Wojtyla et al. (2024) reported that primed seeds improve water uptake and seed imbibition compared to untreated controls. EO treated seeds showed poor germination. SeNPs seed priming also increased the germination percentage of rice seeds compared to untreated controls (Arafa et al. 2024; Yoshikawa et al. 2011). Setty et al. (2023) discovered that priming with SeNPs enhances water uptake in seeds, resulting in the moistening and softening of the seed coat. Seedlings treated with SeNPs exhibited elevated levels of chlorophyll a, b, total chlorophyll content, and chlorophyll stability (%), surpassing those treated with sodium selenite and hydropriming (Table 2). Conversely, seedlings treated with EO showed reduced content at a concentration of 30 ppm. Higher concentrations of EO led to a decrease in dormant cells and an increase in dead cells. An increase in EO concentration resulted in anatomical changes in seedlings, including a decrease in mitochondrial number and damage to the seed coat. Moreover, higher EO concentrations caused structural alterations in root cells, thereby restricting root growth (Yoshimura et al. 2011; Nishida et al. 2005; Bessire et al. 2007). The Membrane Stabilization Index (MSI) measures the percentage of damage or describes the potential of the membrane to survive under unfavorable conditions. In 10 ppm SeNPs seedlings, the membrane stabilization index (MSI) was enhanced up to 94% compared to the positive and negative controls (Sakya et al. 2018).

## 5 Conclusions

The agriculture sector is currently facing challenges such as production losses due to pest attacks, microbial infections, and extreme temperatures. This study introduced EO‐mediated selenium nanoparticles to inhibit the growth of phytopathogens like *Tuta absoluta*, bacterial infections (*X. campesteris* and *P. syringae* ), and fungal infections ( *A. niger* and *F. oxysporum*). The optimized dosage of selenium nanoparticles (10 ppm) significantly affected the tomato seed germination rate, SVI, chlorophyll stability, membrane stability, and root ion leakage, indicating protection against biotic stressors. The study concluded that EO‐mediated SeNPs are a multifunctional tool for sustainable tomato crop management, offering an environmentally friendly, non‐toxic alternative to synthetic chemicals. The findings recommend field trials to validate the effectiveness of SeNPs in combating tomato diseases and improving the yield, nutritional value, and shelf life of tomato fruits in different climatic conditions.

## Author Contributions

**Zainab Maqbool:** investigation (equal), methodology (equal), writing – original draft (equal). **Arusa Aftab:** supervision (equal), visualization (equal). **Humaira Rizwana:** conceptualization (equal), funding acquisition (equal). **Zubaida Yousaf:** data curation (equal), writing – review and editing (equal). **Shazia Rehman:** investigation (equal), software (equal), validation (equal).

## Conflicts of Interest

The authors declare no conflicts of interest.

## Data Availability

All the associated data will be provided upon reasonable request by the corresponding author.
